# A comprehensive study on different modelling approaches to predict platelet deposition rates in a perfusion chamber

**DOI:** 10.1038/srep13606

**Published:** 2015-09-22

**Authors:** Jordi Pallarès, Oriol Senan, Roger Guimerà, Anton Vernet, Antoni Aguilar-Mogas, Gemma Vilahur, Lina Badimon, Marta Sales-Pardo, Salvatore Cito

**Affiliations:** 1 Department of Mechanical Engineering, Universitat Rovira i Virgili, Tarragona, Av Paїsos Catalans 26, 43007, Spain; 2 Department of Chemical Engineering, Universitat Rovira i Virgili, Tarragona, Av Paїsos Catalans 26, 43007, Spain; 3 Institució Catalana de Recerca i Estudis Avançats (ICREA), Barcelona, Passeig Lluís Companys 10, 08010, Spain; 4 Catalan Institute of Cardiovascular Sciences, CSIC-ICCC, Hospital de la Santa Creu i Sant Pau, Barcelona, Sant Antoni Maria Claret 167, 08025 Spain; 5 University of Helsinki, Faculty of Pharmacy, Helsinki, Viikinkaari 9 00014, Finland

## Abstract

Thrombus formation is a multiscale phenomenon triggered by platelet deposition over a protrombotic surface (eg. a ruptured atherosclerotic plaque). Despite the medical urgency for computational tools that aid in the early diagnosis of thrombotic events, the integration of computational models of thrombus formation at different scales requires a comprehensive understanding of the role and limitation of each modelling approach. We propose three different modelling approaches to predict platelet deposition. Specifically, we consider measurements of platelet deposition under blood flow conditions in a perfusion chamber for different time periods (3, 5, 10, 20 and 30 minutes) at shear rates of 212 s^−1^, 1390 s^−1^ and 1690 s^−1^. Our modelling approaches are: i) a model based on the mass-transfer boundary layer theory; ii) a machine-learning approach; and iii) a phenomenological model. The results indicate that the three approaches on average have median errors of 21%, 20.7% and 14.2%, respectively. Our study demonstrates the feasibility of using an empirical data set as a proxy for a real-patient scenario in which practitioners have accumulated data on a given number of patients and want to obtain a diagnosis for a new patient about whom they only have the current observation of a certain number of variables.

Thrombosis is the main responsible for the leading causes of mortality and morbidity worldwide: heart attack and ischemic stroke^1^. Thrombus formation is an extremely complex pathological process that starts upon platelet interaction with the exposed vascular thrombogenic surface upon atherosclerotic plaque rupture. Concomitantly, tissue factor exposure triggers the activation of the coagulation cascade and thrombin formation further promoting platelet activation and aggregation. Thrombin, in turn, also leads to fibrin formation and thrombus stabilization.

Experimental evidence shows that platelet activation and deposition depends on hemodynamic and rheological variables such as shear rate, shear stress^2^, red blood cell margination^3^,^4^, exposed substrate (subendothelium, collagen, tendon, etc.) and local concentration of activated platelets and pro-thrombotic factors^5^,^6^. Despite the development of several theoretical models that describe the many contributors to thrombus formation and growth^7^, with special emphasis on the platelet aggregation process^3^,^8^,^9^,^10^,^11^,^12^ as well as the spatial and temporal aspects of early stage thrombus dynamics^13^, the role of each of the aforementioned variables on thrombus formation is still not clear thus hindering the development of comprehensive and computationally fast multiscale models^14^,^15^,^16^.

In view of this challenge, and as a first step towards the understanding of the role and limitations of different modelling approaches for thrombus formation, our goal is to compare distinct computationally fast approaches to predict platelet deposition levels. While platelet deposition has been extensively studied, especially within the hemodynamics literature^17^,^18^,^19^,^20^, very little emphasis has been placed on the assessment of the predictive power of such models. Specifically on the evaluation of whether models adjusted to a set of empirical data (training data set) provide a good description of a different empirical data set (test data set). To a large extent, this is due to the lack of extensive, systematic empirical data on platelet deposition for a wide range of experimental conditions.

To cover this gap, we analyze the ability of different computational approaches to predict platelet deposition values for a large variety of empirical conditions. Note that as a first step, we focus on total platelet deposition counts and do not take into account the spatial dimension of thrombus formation^13^. Specifically, we consider the following approaches: a) a mechanistic modeling approach, b) a machine learning approach; and c) a phenomenological approach. We find that a phenomenological approach built upon empirical facts of the platelet deposition process has the largest predictive power thus offering novel insights into what are the effective roles of different blood factors in platelet deposition.

**Approach and rationale.** Figure 1 illustrates the approach we followed in our study. Specifically, we first collected the platelet deposition data. Then, in order to asses the predictive power of the different computational approaches, we performed a cross-validation analysis. In this type of analysis, we divide the collected data into a training dataset and a test dataset. We use the training dataset to train our model or algorithm (that is to obtain model parameters) so that we obtain a good agreement between model/algorithm outputs and the known empirical platelet deposition value. Then, for each experimental condition in the test dataset, we use the trained model/algorithm to make a prediction of the platelet deposition value. We compare the predicted value with the real value obtained from the experiments to assess the error of the prediction of each approach.

**Experimental data collection.** In our analysis, we consider platelet deposition data of pig blood obtained using a validated *ex vivo* perfusion chamber (Badimon chamber^5^,^21^), (see Methods). The Badimon chamber provides an excellent proxy for the patho-physiological environment that affects platelet deposition because: i) it is a bio-reactor that retains the cylindrical shape of vascular conduits in which one can simulate a broad range of flow conditions^21^,^22^; ii) it is flexible enough to test the thrombogenicity associated with different vascular surfaces or atherosclerotic lesions^23^; and, iii) it allows to analyze different blood conditions and blood treatments^24^,^25^. Specifically, we obtain platelet deposition data for four different pigs under a number of different experimental conditions including variation in shear rate, perfusion time, vascular tissue, hematocrit and platelet concentration levels (see Table 1 for a summary of the collected data).

**Computational approaches.**We consider three complementary computationally fast approaches to model platelet deposition (see Fig. 1 for a summary of the main advantages and limitations of each approach):

- (a) A novel mechanistic model based on the mass-transfer boundary layer theory (MBL)^26^. This is an approach that has been extensively used to investigate hemodynamics and platelet deposition in particular^8^,^10^,^27^,^28^,^29^,^30^,^31^. This type of models assume that the platelet deposition rate is proportional to a reaction kinetics constant and to the platelet concentration at the wall^8^,^10^. We consider a generalization of a simple model of platelet deposition that includes implicitly the effect of the convective force using boundary-layer theory and as a novelty differentiates between the first monolayer of platelet deposition [platelets in contact with the substrate (e.g. endothelial layer)] and the following multi-layer platelet aggregates [platelet-platelet interaction and thrombus growth] (see Methods and Supplementary Material). As a result, the number of deposited platelets depends on the platelet and hematocrit levels in blood, the vascular lesion dimensions and two kinetic reaction constants that need to be determined: *k*_1_ for the formation of the first monolayer and *k*_2_ for the formation of subsequent layers (see Methods). Note that within our approach deposited platelets cannot detach.

The MBL approach has the advantage that it provides a mechanistic description of the platelet deposition process in which parameters have a clear physical meaning. However, due to MBL assumptions its application is limited to experiments with no stenosis (since the flat plate boundary layer assumptions would be violated) and for short perfusion times (see Methods).

- (b) A machine-learning approach using the Random Forest algorithm (RF)^32^. Methods such as the RF^32^ are especially suited to predict the outcome (for instance, number of deposited platelets) of an event given the observation of certain features (such as the hematocrit level, shear rate and platelet concentration), without a priori knowledge of the mechanisms governing the specific phenomenon. Indeed, the RF has been successfully applied in a variety of biological contexts such as protein interaction prediction^33^, gene classification^34^ and feature selection in biological models^35^.

Importantly the RF can process both qualitative and quantitative variables, which make it suitable for our analysis in which we have both types of variables (e.g. vascular tissue and blood type are qualitative, while the remaining variables are quantitative—see Table 1). However, the predictive power of the RF is severely affected by the range of the training dataset, and will produce very bad predictions for any new input data that falls out of that range.

- (c) A phenomenological model (PM) constructed from empirical evidences collected in platelet deposition experiments. We consider a model that takes into account the a priori most relevant features, based on the following observations from the empirical data and from the literature, and further refined with the analysis of variable importance using the RF (see Supporting Figure S1-3):

- Platelet deposition counts increase, in general, with perfusion time and show no apparent signs of saturation in the measured times (see Supporting Figure S1-4);
- Platelets cannot deposit on a surface if there are no platelets circulating in blood;
- Tissue type affects the rate of platelet deposition^2^,^31^,^36^;
- The shear rate affects the rate at which platelets deposit on a surface^8^,^21^,^37^.

Taking into account these simple facts, we propose the following phenomenological model for the logarithm of the total platelet deposition *P* under certain experimental conditions:


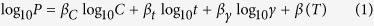


where *P* is the platelet accumulation, *C* is the platelet concentration in blood, *t* is the perfusion time, *γ* is the shear rate, {*β*_*C*_, *β*_*t*_, *β*_*γ*_} are constants, and *β*(*T*) is a constant that depends on the vascular tissue type (therefore it takes 3 different values).

Our cross validation analysis reveals that the PM has a larger predictive power than MBL and RF approaches: average median errors of 21% (MBL), 20.7% (RF) and 14.2% (PM).

## Results

### Model validation

We first assess the validity of the three approaches we consider by fitting the models to all available data points. Figure 2 shows that the three approaches we propose—(a) MBL, (b) RF, (c) PM—are, in principle, suited to obtain accurate platelet deposition values under different empirical conditions. The fitting parameters for the MBL model are the kinetic constants of the platelet adhesion process on the substrate (*k*_1_) and on a layer of a previously deposited platelets (*k*_2_). The PM has four fitting parameters: *β*_*C*_, *β*_*t*_, *β*_*γ*_ and *β*(*T*), associated, respectively, to the platelet concentration in blood, the perfusion time, the shear rate and the substrate. The top rows in Tables 2 and 3 show the model parameters estimated for MBL and PM approaches, respectively.

In the MBL approach, we find that platelet deposition counts on tunica media corresponding to a severely damaged vessel wall in which deeper vascular layers are exposed (i.e., vascular smooth muscle cell), does not depend on the values of *k*_1_ and *k*_2_. This suggests that for the experimental conditions under consideration, the deposition on this substrate was limited by the advective and diffusive transport of platelets towards the wall. For the other two substrates (pig tendon and subendothelium), we find that *k*_1_ and *k*_2_ are roughly independent of the tissue and that the values of *k*_2_ are about one order of magnitude larger than *k*_1_. This is consistent with the fact that in the PM (Table 3) we obtain the same value for the tissue parameters corresponding to subendothelium and pig tendon and a different value for tunica media.

This observation agrees with the expectation that platelet deposition occurs in a similar manner on both substrates because of their similar constituents. Pig tendons are a rich source of collagen fibers which are precisely one of the main constituents of the basal membrane, the layer that is exposed (but not damaged) in a subendothelial exposure. On the other hand, tunica media encompasses endothelial denudation with damage to both intima and the vascular media exposing to the blood flow not only collagen proteins but vascular smooth muscle cells and their constitutive proteins. Such proteins are highly thrombogenic^5^ and therefore affect differently the platelet deposition process.

### Predictive power assessment

**Figure 1:**
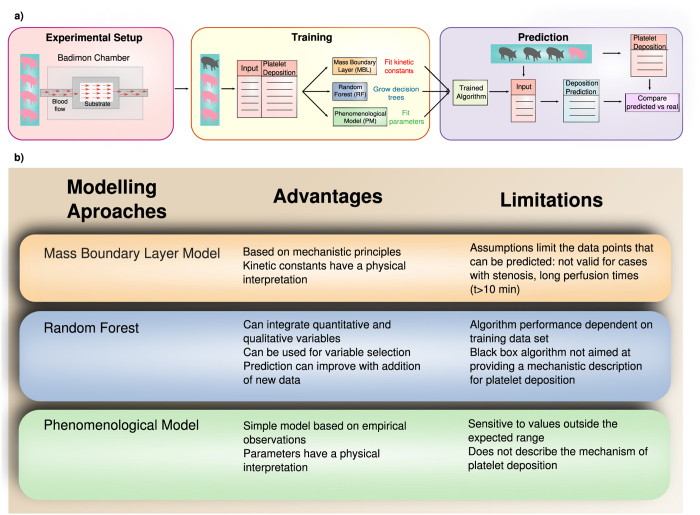
Flowchart and summary of our approach. (**a**) Flowchart of the analysis. Our study is divided in three steps: i) experimental setup and data collection; ii) training of models/algorithms; iii) prediction. *Experimental setup and data collection:* In the experiments, pig blood circulates from the animal to a perfusion chamber (Badimon Chamber) containing one of the three different vascular tissues considered triggering thrombi (tunica media, pig tendon, subendothelium). We collected platelet deposition counts for different experimental conditions such as perfusion time or shear rate (see Table 1 and Methods). We performed experiments with four different animals. *Training:* We consider all the collected input (experimental conditions) and corresponding platelet deposition data for three pigs. With this information we train the models/algorithms to get a good agreement between model/algorithm outputs and known platelet deposition values. *Prediction:* We now consider the data collected for the remaining pig. We use the experimental conditions in that dataset as inputs to the trained model/algorithm to obtain predictions of platelet deposition values for each set of conditions. We test the prediction power of each model/algorithm by comparing predicted platelet deposition values to measured platelet deposition values. We carry out steps ii) and iii) for the four different combinations of training (3 pigs) and test (1 pig) datasets. (**b**) Advantages and limitations of each of the computational approaches for platelet deposition prediction that we consider in our study: a mass-transfer boundary layer model, the Random Forest algorithm and a phenomenological model (see text).

**Figure 2:**
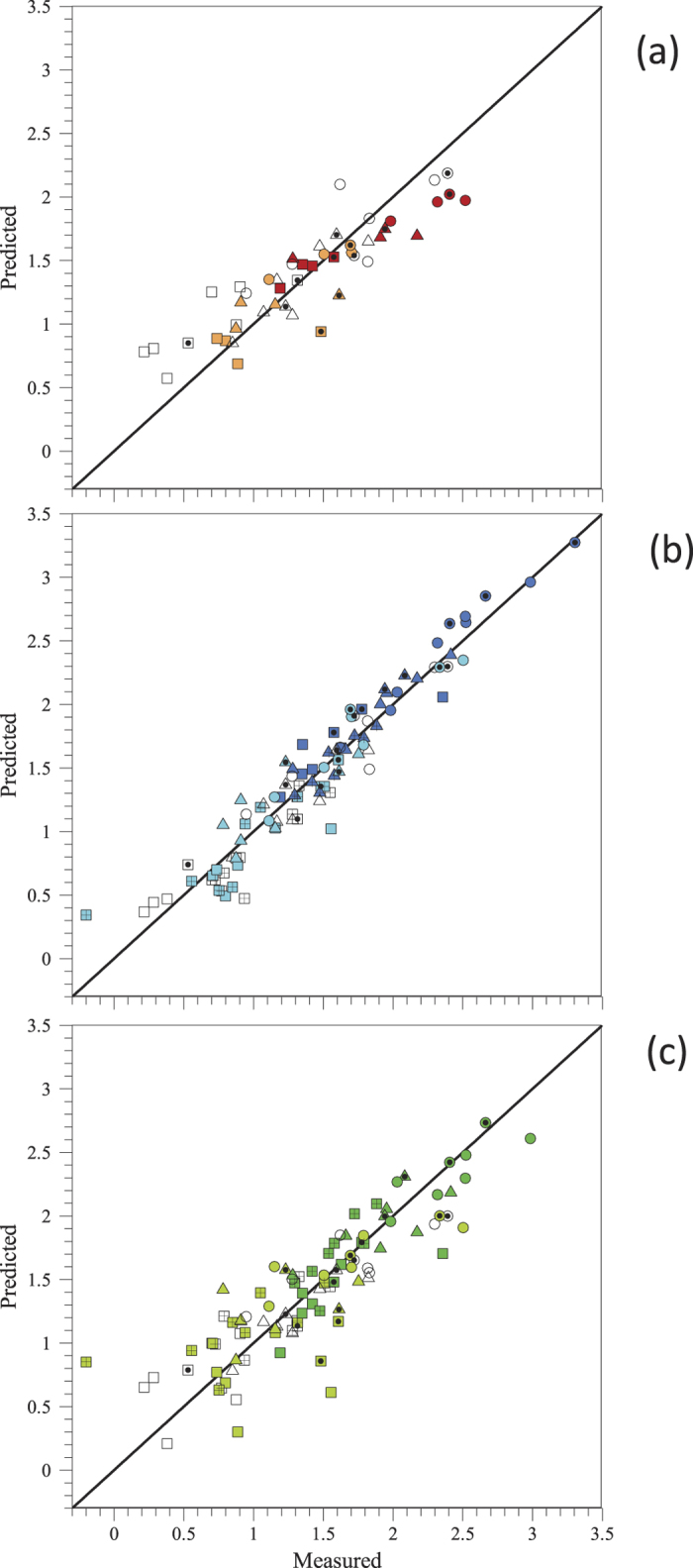
Platelet deposition predicted by (**a**) the mass-boundary layer model (MBL) (**b**) Random Forest (RF) and (**c**) the phenomenological model (PM). We show the predictions as log_10_(number of platelets/cm^2^ × 10^−6^) versus the corresponding experimental values. Open symbols correspond to a perfusion time of 3 minutes, light color symbols to 5 minutes and dark color symbols to 10 minutes. Symbols with a cross represent data of native blood, symbols with dots and without dots correspond to different concentration of heparin (35 + 35U/K/H and 120 + 100U/K/H, respectively).

**Figure 3:**
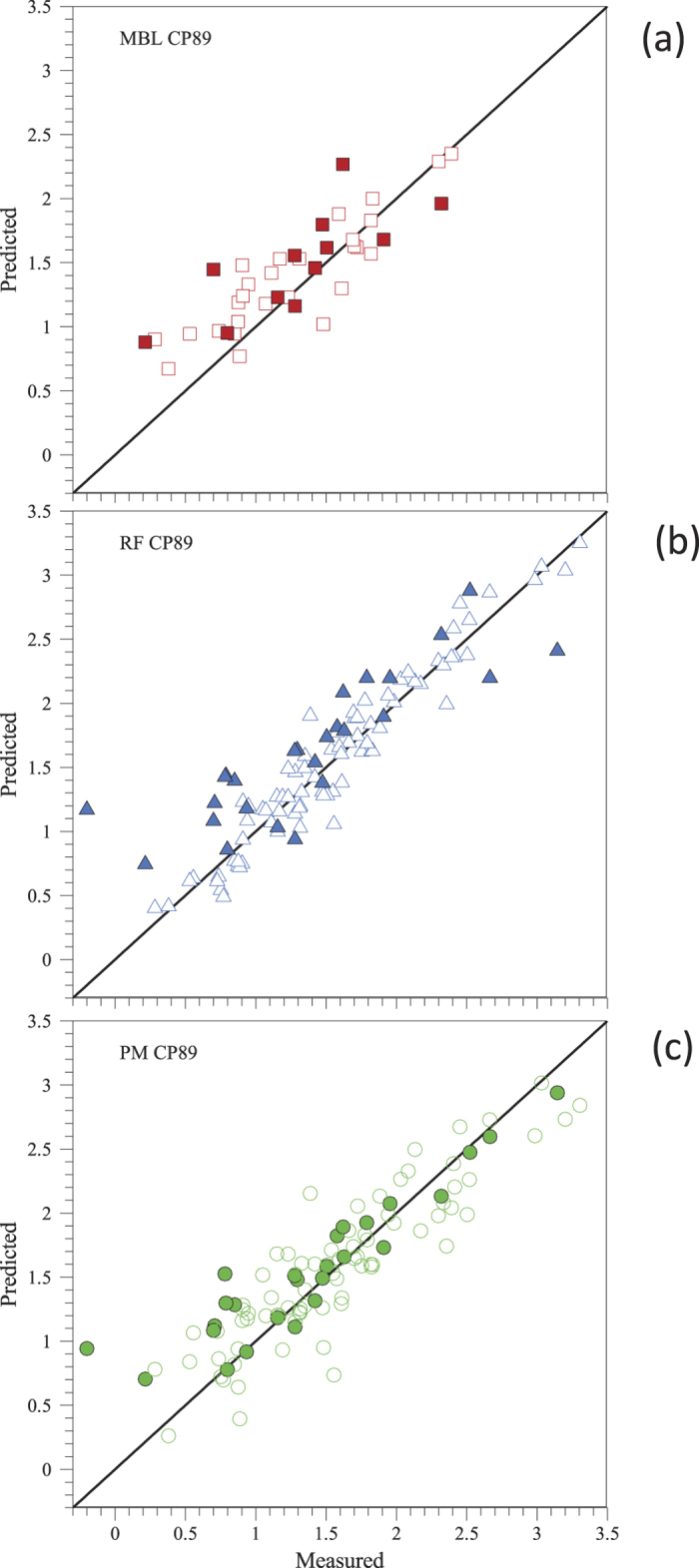
Cross-validation plot for pig CP89 showing platelet deposition predicted by (**a**) the mass-boundary layer model (MBL, red squares), (**b**) Random forest (RF, blue triangles) and (**c**) the phenomenological model (PM, green circles). We show model predictions as log_10_(number of platelets/cm^2^ × 10^−6^) versus the corresponding experimental values for which MBL can produce a prediction (no stenosis). Open symbols correspond to the training set and filled symbols correspond to the test set. Parameters for PM: *β*(*T*) = −6.3 (PT), −6.3 (SE), −5.8 (TM), *β*_*C*_ = 2.2, *β*_*t*_ = 1.33, *β*_*γ*_ = 0.402.

In order to assess the predictive power of each one of the approaches, we performed four cross-validation experiments (Fig. 1). In each one of these experiments, we consider three pigs as the ‘training’ data set, and the remaining pig as our ‘test’ data set. Therefore, we use data from three pigs to estimate the kinetic constants in the MBL approach (see Table 1), to train the RF and to estimate the parameters in the PM (see Table 2). We then evaluate the error of each of these three approaches in predicting platelet deposition values for the remaining pig. Figure 3 shows, as an example, the cross-validation plot for pig CP89.

**Figure 4:**
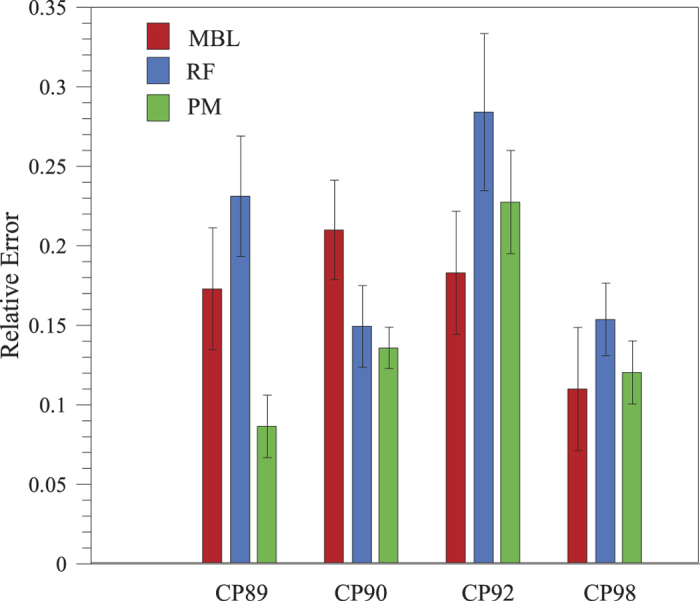
Median relative error in the test sets. For each one of the cross-validation analysis we show the median relative error: difference between the predicted and the measured value, relative to the measured value. Error bars correspond to median absolute deviation divided by the square root of observations. For each one of the approaches: MBL—Mass Boundary Layer Model, RF—Random Forest and PM—phenomenological model.

Our analysis shows that the three approaches we propose produce reasonable predictions of the amount of deposited platelets (Fig. 4). Note that we can build further confidence in the MBL and PM because model parameters show little variation (that is, are always in the same orders of magnitude) across the set of cross-validations. We note that in the PM all parameters in Eq (1) are significantly different from zero. In addition, in the case of pig tendon and subendothelium, the tissue parameters (*β*(*T*) in Eq (1)) are very similar, confirming that there is little difference in platelet deposition on these two substrates as expected.

**Figure 5:**
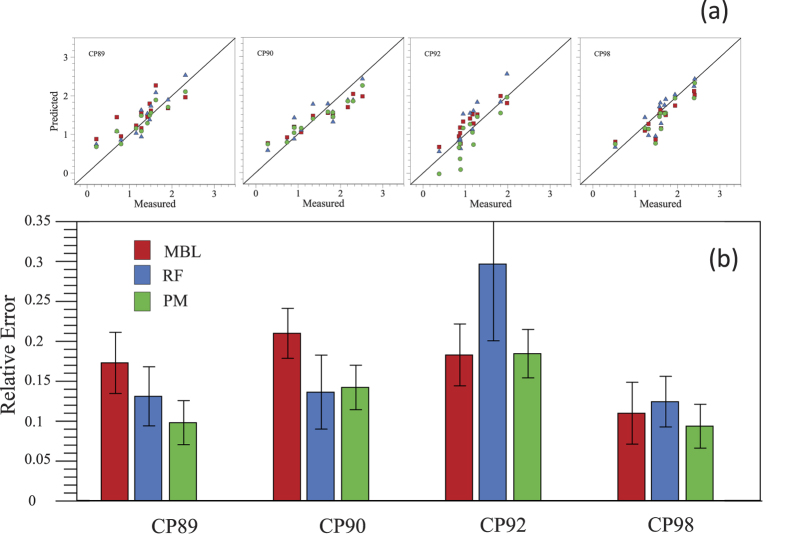
(**a**) Prediction and true value of platelet deposition of test sets. We use the same data points in each test set to directly compare the three modelling methods. (**b**) Median error of previous cross-validation, relative error: difference between the predicted and the measured value, relative to the measured value. Error bars correspond to median absolute deviation (MAD) divided by the square root of observations. MBL: Mass Boundary Layer (squares) Model, RF = Random Forest (Triangles), PM = Phenomenological Model (circles).

In order to quantify the predictive power of each one of the approaches, we compute the relative error for each one of the cross-validations performed with the three approaches (Fig. 5 and Supporting Figure S1-5). We note that the median error is typically low, and that the PM is the model that performs best. On average the PM shows relative errors typically about 14.2%, while MBL and RF approaches have median errors of 21% and 20.7%, respectively. This is also the case if we only consider data points for which MBL can produce predictions (that is, experiments with no stenosis), for which the PM has an average median error of 12.9%, while MBL and RF approaches on average have median errors of 22% and 17.2%, respectively.

We also note that in one of the cases (when predicting platelet deposition for pig CP92) we find that the RF and PM approaches have a much lower predictive power. An inspection of the data reveals that this dataset has a narrow range of platelet deposition values—CP92 platelet deposition: (platelets/cm^2^ × 10^−6^) [2.4, 135.3]—, while the rest of data has a wider range—[0.62, 2013.74] (platelets/cm^2^ × 10^−6^)—and that values are lower for CP92 ([182.0, 287.07] (platelets/*μ*l × 10^−3^) than for the other three pigs (platelet concentration [289.07, 498.89] (platelets/*μ*l × 10^−3^). Therefore, the loss of predictive power is probably due to the fact that the training data set has ‘less’ information in the region where CP92 points lie since the training set covers a broader range. This issue highlights the importance of the training set in order to obtain accurate predictions.

## Discussion

Our study showcases the validity of computational approaches to predict platelet deposition in vascular tissues in a number of different conditions. First, we empirically assessed platelet deposition exposing animal blood to a thrombus triggering substrate during different time periods and at different shear rates. Then, we tested the predictive power of three complementary approaches: i) a principle based approach using a mass-transfer model; ii) a machine learning approach that has no information about the physico-chemistry behind the biological process (Random Forest); iii) a phenomenological model constructed from empirical evidence.

Our study shows that the three approaches have a consistent predictive power, the phenomenological model having an overall better performance. Furthermore, our analysis highlights the main advantages and disadvantages of the different approaches (see Fig. 1).

Our analysis also shows that RF and PM approaches would significantly benefit from the availability of platelet deposition data for a larger variety of empirical conditions (for instance, different shear rates and perfusion times). However, this is not necessarily the case for the MBL model. The assumptions made in such model impose certain limitations on the range of applicability of the model. In particular, our MBL approach is not applicable to cases with stenosis or for long times of perfusion when platelet detachment may occur (see for example Supporting Figure S1-4c, where a decrease of deposited platelets is observed for perfusion times between 10 and 30 minutes). The extension of the range of applicability of the MBL model to these cases would require to take into account and parametrize a) the variation of the wall shear rate along the substrate with stenosis and b) the mechanisms responsible for the platelet detachment, thus entailing an increase in the number of fitting parameters.

The availability of a larger variety of empirical conditions would help improve the prediction power of the PM in two aspects. One the one hand, it would yield a more robust set of model parameter values that would give good predictions for a larger range of empirical conditions. On the other hand, new experimental data could help uncover new empirical facts that could be used to refine our model.

Finally, our study shows that the parameter based approaches we propose are biologically sound. Remarkably, our mass-transfer model is a novel model that built upon common approaches in literature that explicitly differentiates between the formation of the first monolayer and that of the subsequent layers. The fact that the kinetic constants associated to each of these mechanisms are different by an order of magnitude indicates that this is an important aspect of the platelet deposition process. In the PM, the fact that all the model parameters are different from zero all the variables we selected have a distinct impact in the platelet deposition process. Additionally, for both approaches we obtain parameter values that are consistent with our expectation of the differences of deposition on different substrates. In particular, in the PM approach tissue dependency is well captured by a single parameter that is similar for pig tendon and subendothelial tissues and different for the tunica media. In contrast, the parameters associated to shear rate, platelet concentration in blood, and perfusion time remain the same throughout the analysis. In fact, according to Table 3 the largest contribution is that of platelet concentration in blood and perfusion time, which is also consistent with the assumptions in the MBL model.

All in all, our study opens the door toward further studies that aim to integrate macroscopic description of the models we propose by coupling it to more refined models of the microscopic processes behind platelet deposition.

## Methods

### Data description and prediction experiments

#### Experimental animal model

Experiments were performed in Large White x Landrace commercial pigs (n = 4, m ≈ 36 kg), individually caged in a light-, temperature-, and humidity-regulated environment with controlled feeding and free access to water. The investigation conforms to the Guide for the Care and Use of Laboratory Animals published by the US National Institute of Health (NIH Publication No. 85-23, revised 1996).

#### Radioactive labeling of platelets

We performed radioactive labeling of platelets to monitor their deposition (monolayer and multilayer). To that purpose, after overnight fasting, 43 ml of pig blood was drawn in 7 ml of anticoagulant citrate dextrose solution by femoral venipuncture. Platelets were isolated and labeled with ^111^In (Amersham Biosciences, UK) as described in^25^ suspended in a final volume of 4 ml of autologous plasma, and reinjected into the pig (ear vein) within 2 h. Labeling efficiency was around 90% and the injected activity was around 250 microCi. Post-mortem ^111^In biodistribution indicated a correct platelet distribution with maximal accumulation in blood.

#### Extracorporeal perfusion system in the Badimon chamber

The study protocol was approved by the institutional ethics committee (CSIC-ICCC) and all animal procedures were performed conform the guidelines from Directive 2010/63/EU of the European Parliament on the protection of animals used for scientific purposes or the NIH guidelines. In addition, we have followed the ARRIVE guidelines^38^. We assessed platelet behavior by exposing the animal blood to a thrombus triggering substrate during different time periods and at different shear rates in the previously validated and standardized Badimon perfusion chamber^21^. To that end, after overnight fasting, animals were tranquilized (8 mg kg^−1^ Stressnil, Esteve), anesthetized (10 mg kg^−1^, B. Braum, Spain), and a carotid artery-jugular vein shunt was established to place the Badimon perfusion chamber as previously described^25^. All of the animals received low-dose anticoagulation with heparin (50 IU kg^−1^) as a continuous infusion to avoid clotting inside the tubing system. This heparin regime does not affect platelet deposition^21^.

Blood was perfused through the chamber for different time periods (3, 5, 10, 20 and 30 minutes) at shear rates of 212 s^−1^, 1690 s^−1^ and at an experimental stenosis of 80%, that corresponds to a shear value of 1390 s^−1^, in order to mimic the rheological conditions within blood vessels (see the following section for details on the calculation of these values). The thrombogenic substrates (platelet-triggering surfaces) included homologous porcine vessel walls with 2 types of damage [mild (denuded vessel wall or subendothelium SE) and severe (disrupted vessel wall or tunica media TM)] and pig tendon (PT). Several perfusions with varying time of perfusion, hemodynamic conditions and triggering substrate were performed in each animal. After the perfusion, vessels were fixed in 4% paraformaldehyde to count labelled platelets using a gamma counter (Wizard, Wallac, USA). Values were normalized by blood ^111^In activity (counts), platelet counts in blood, and area exposed surface^25^. At the end of the experiment, animal’s heart was arrested with a 10 ml potassium chloride 2M intravenous injection.

#### Hematological and hemodynamic parameters

We determined hematocrit and platelet count throughout the experimental period with as System 9000 Serono cell analyzer.

#### Overview of the data

Table 1 provides an overview of the type and range of data collected from the experiments.

For the perfusions performed with 80% of stenosis, we computed the shear rate solving numerically the Navier-Stokes equations in the three dimensional domain that emulate the perfusion chamber with and without the stenosis (see S3 for details).

An analysis of the empirically measured platelet deposition counts reveals that the distribution of the logarithm of the number of deposited platelets has no gaps and is smoother than the distribution of the number of deposited platelets (see Figure. S1-1). For this reason, we focus on predicting the log_10_ of the number of deposited platelets.

### Computational approaches to platelet deposition

#### Mass-transfer boundary-layer model (MBL)

Convection-diffusion-reaction models assume that the platelet deposition rate is proportional to a reaction kinetics constant and to the platelet concentration at the wall^8^,^10^,^27^,^28^,^29^,^30^,^31^,^39^,^40^,^41^,^42^,^43^. In here, we consider a generalization of a simple model of platelet deposition that includes implicitly the effect of the convective force using boundary-layer theory and differentiates between the first monolayer of platelet deposition [platelet in contact with the substrate (e.g. endothelial layer)] and the following multi-layer platelet aggregates (platelet-platelet interaction and thrombus growth).

Specifically, in our model we assume two different kinetic reaction constants: *k*_1_ for the formation of the first monolayer and *k*_2_ for the formation of subsequent layers. Therefore, we consider that as the first layer is being covered, with a maximum number of platelets 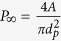 where *A* = *δW* is the area of the substrate and *d*_*p*_ = 2 × 10^−6^ m is the diameter of an adhered platelet^10^, the second layer starts to form. We model the two adhesion processes with first order kinetics.

In our model, for each one of the layers *i* we consider, the platelet deposition rate 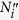 given certain wall flux of platelets depends on the available deposition area *WL*_*i*_,


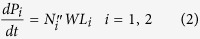


with 
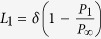
 and 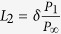


We assume that the diffusion, advection and reaction processes occur within a two-dimensional mass transfer boundary layer much thinner than the diameter of the perfusion chamber; and that there is a defect of concentration of platelets in comparison with the bulk concentration in the blood (see Supporting Material S2 for a full derivation and for a discussion about the physical interpretation of the equations), the platelet flux on a substrate of length *L* can be written as^26^ (see Supporting Material S2),


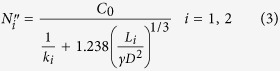


where *C*_0_ is the bulk concentration of platelets in the blood flow, *γ* is the shear rate, which is assumed to be constant within the mass transfer boundary layer thickness and *D* is the diffusion coefficient that depends on the hematocrit concentration^44^ (see Supporting Material S2).

To numerically determine the kinetic constants using the MBL model, we assume that *k*_1_ depends only on the type of substrate used in the experiments. For each set of experiments with a given substrate, we then compute the time evolution of *P*_1_ and *P*_2_ (see Eqs. S2-10 and S2-11). We then perform the calculations for several values of *k*_1_ and *k*_2_ in the ranges 10^−3^ ≤ *k*_1_ ≤ 10^−8^ m/s and 10^−3^ ≤ *k*_2_ ≤ 10^−8^ m/s. For each pair of values (*k*_1_, *k*_2_), we then compute the absolute difference between the predicted value of the total number of platelets deposited and the corresponding experimental value at a given time. For each different substrate, we select the pair of values (*k*_1_, *k*_2_) that minimizes the absolute difference between the measured and predicted values.

### Random Forest (RF)

We use Random Forest to predict the log_10_ of the platelet deposition count using four quantitative features and two qualitative features (see Table 1). In our analysis, we used the Random Forest Package version 4.6–7^45^ within R version 3.0.2^46^. We set the algorithm to the following parameters (mtry = 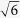, ntree = 1000). In order to control for the slight variation of each forest due to the bagging process, we performed 100 times each RF. For the estimation of the feature importance, we leaved one feature out of the Random Forest and computed the error rate. Additionally, we applied a linear correction to initial RF predictions to improve the error rate (see Supporting Figure S1-2).

### Phenomenological model for platelet deposition (PM)

We estimate the parameters by performing a least-squares fit of the data using the R software^46^.

## Additional Information

**How to cite this article**: Pallarès, J. *et al.* A comprehensive study on different modelling approaches to predict platelet deposition rates in a perfusion chamber. *Sci. Rep.* **5**, 13606; doi: 10.1038/srep13606 (2015).

## Supplementary Material

Supplementary Information

## Figures and Tables

**Table 1:** Experimental data.

Variable	Values (Mean, range)
Shear rate (s^−1^)	212, 1390 and 1690
Perfusion time (min)	3, 5, 10, 20 and 30
Hematocrit (%)	Mean: 26.46 (PCV), [22.0, 31.30]
Platelet concentration (platelets/*μ*l) × 10^−3^	Mean: 341.096, [182.0, 449.0]
Blood	Native blood and heparinized blood
Vascular tissue	PT—pig tendon; TM—tunica media; SE—subendothelium
**Platelet deposition (platelets/cm^2^ × 10^−6^)**	**Mean: 130.68 [0.63, 2013.5]**

**Table 2:** MBL model parameters.

Test	*k*_1_ (PT)	*k*_2_ (PT)	*k*_1_ (SE)	*k*_2_ (SE)
(1 pig)	(m/s) × 10^7^	(m/s) × 10^5^	(m/s) × 10^7^	(m/s) × 10^5^
—	9.5 (0.3)	5.4 (0.4)	8.7 (0.3)	20.0 (0.4)
CP89	10.0 (0.3)	18.0 (0.4)	12.0 (0.3)	13.0 (0.4)
CP90	6.6 (0.3)	5.9 (0.4)	13.0 (0.3)	1.0 (0.4)
CP92	10.0 (0.3)	16.0 (0.4)	12.0 (0.3)	7.5 (0.4)
CP98	6.6 (0.3)	7.2 (0.4)	7.1 (0.3)	9.8 (0.4)

The top row shows the values for *k*_1_ and *k*_2_ obtained considering all the available data for which the model can produce a prediction (no stenosis). The remaining rows show the values obtained for the cross-validation analysis. PT—pig tendon; SE—subendothelium.

**Table 3:** PM parameters.

Test	*β*_*C*_	*β*_*t*_	*β*_*γ*_	*β*(*T*)
—	2.2(0.3)	1.4(0.1)	0.38(0.07)	−6.4(0.8) (PT)−6.7(0.8) (SE)−5.3(0.8) (TM)
CP89	2.2(0.3)	1.3(0.1)	0.42(0.08)	−6.4(0.8) (PT)−6.3(0.8) (SE)−5.8(0.8) (TM)
CP90	2.1(0.3)	1.3(0.1)	0.30(0.09)	−5.7(0.9) (PT)−5.8(0.9) (SE)−5.2(0.9) (TM)
CP92	2.6(0.5)	1.7(0.1)	0.40(0.08)	−8.0(1.0) (PT)−8.0(1.0) (SE)−7.0(1.0) (TM)
CP98	2.0(0.4)	1.3(0.1)	0.40(0.09)	−6.0(1.0) (PT)−6.0(1.0) (SE)−5.0(1.0) (TM)

The top row shows the values [value (error)] for *β*_*C*_ (platelet concentration), *β*_*t*_ (perfusion time), *β*_*γ*_ (shear rate) and *β*(*T*) (tissue) obtained considering all the available data. The remaining rows show the values obtained for the cross-validation analysis considering data for the specified pig as the test set and data for the remaining pigs as the training set. PT—pig tendon; SE—subendothelium, TM—tunica media.
