# Supplementary material for: A comprehensive study on different modelling approaches to predict platelet deposition rates in a perfusion chamber
Source: Sci Rep. 2015 Sep 22;5:13606. doi: 10.1038/srep13606 (PMC4585733; doi:10.1038/srep13606)
Supplement: Supplementary Information [file srep13606-s1.pdf]

Supporting Material for:A comprehensive study on different modelling approaches to predict platelet deposition rates in a perfusion chamber

Jordi Pallarès[1]*, Oriol Senan[1]*, , Roger Guimerà[1,3], Anton Vernet[1], Antoni Aguilar-Mogas[1], Gemma Vilahur[4], Lina Badimon[4], Marta Sales Pardo[1]†, Salvatore Cito[1,2]

Table of contents

S1: Supporting Figures

S2: Multi-layer mass transfer boundary layer model (MBL)

S3: Calculation of shear rate in experiments with 80% of stenosis

*These authors contributed equally to this work

†Corresponding author. E-mail: marta.sales@urv.cat (MS)

S1 Supporting Figures

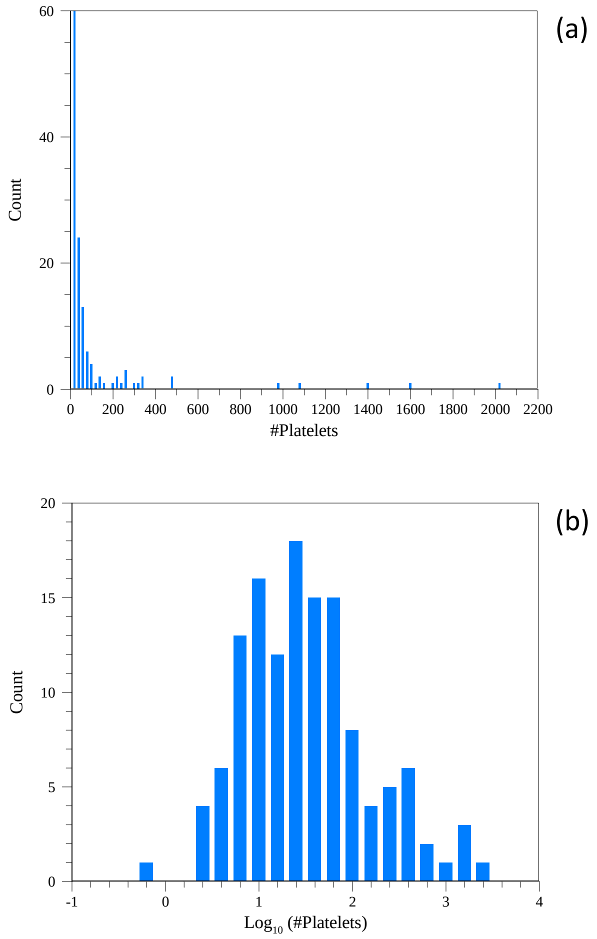

Figure S1- 1: Distribution of the number of deposited platelets. (a) Distribution of the number of platelets (cm^{-2} 10^{-6}) deposited for all empirical conditions. Note how we observe a few values that have much larger platelet deposition numbers. (b) Distribution of the logarithm of the number of deposited platelets ($\log_{10}$ (number of platelets) cm^{-2} 10^{-6}) for all empirical conditions we study. It is obvious that the distribution of the logarithm is fairly narrow and shows no gaps suggesting that this is a more suitable variable for prediction.

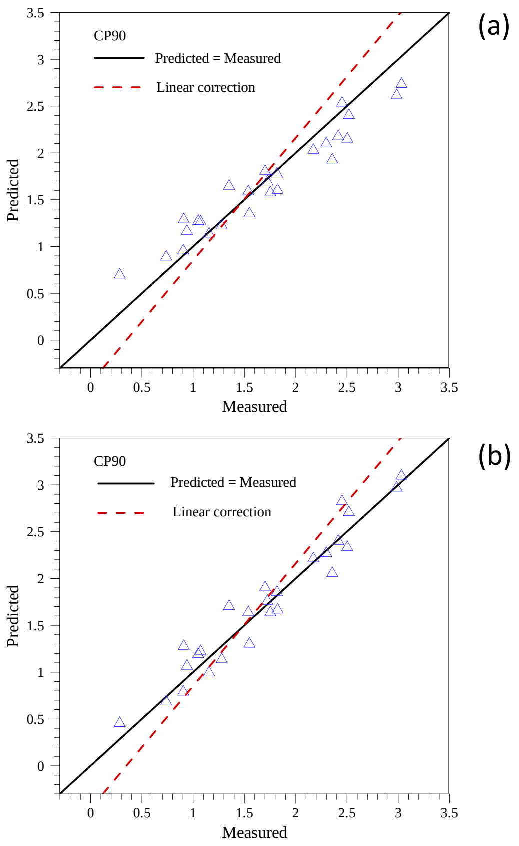

Figure S1- 2: Random Forest (RF) linear correction for the data of pig CP90. Scatter plot of predicted value of platelet deposition versus the measured value, in a logarithmic scale as the logarithm of the number of deposited platelets ($\log_{10}$ (number of platelets) cm^{-2} 10^{-6}). The black line is the line with null error, used for reference purposes. (a) RF prediction before the linear correction, for pig CP90. Dashed red line corresponds to the linear fit, with regression coefficient 1.31 and y-intercept 0.456, r^2=0.9992, p-value=$1.2{\cdot}10^{-6}$. (b) Predicted platelet deposition values obtained after substituting the RF prediction values in the linear regression equation as independent variables.

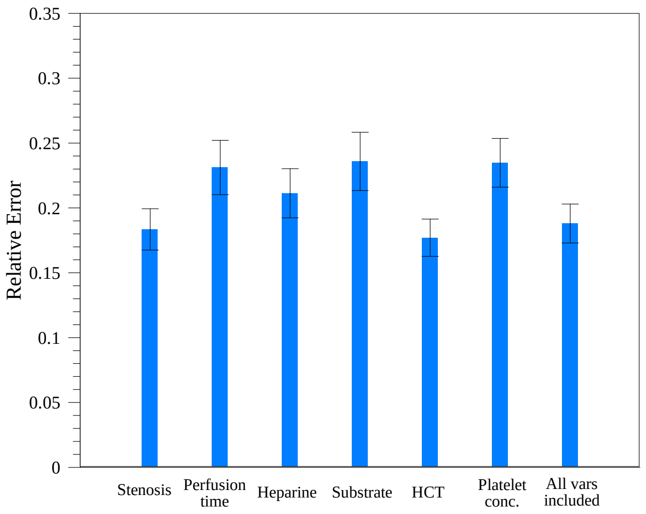

Figure S1- 3: Importance of each feature on the prediction of the platelet deposition using the Random Forest algorithm. Bars depict the relative error in the prediction when the labeled feature is not used. For comparison purposes a bar with the relative error committed when using all features is also included. The confidence intervals represented correspond to the median absolute deviation (MAD) divided by the square root of the number of observations.

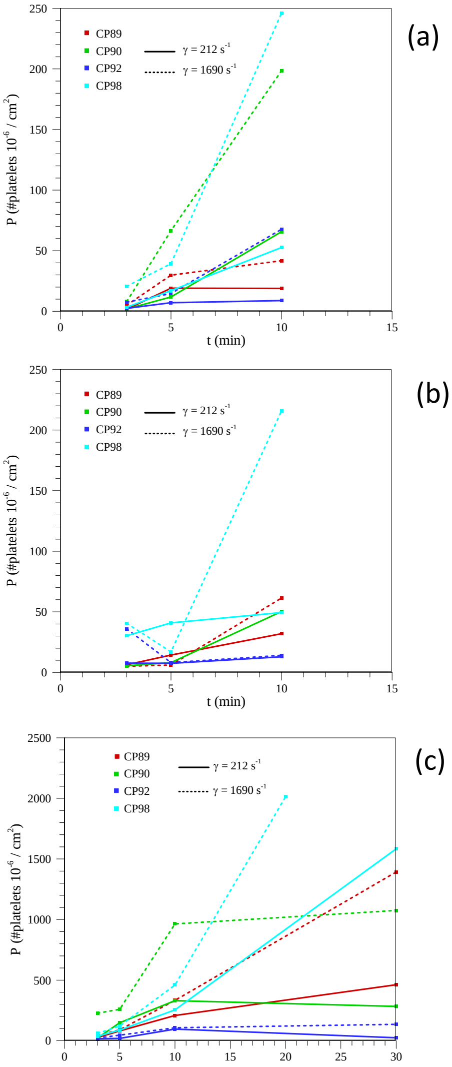

Figure S1- 4: Evolution of platelet deposition over time in the different substrates. (a) Pig tendon, (b) subendothelium, (c) tunica media. Colors indicate the pig from which the measurements come (red for CP89, green for CP90, blue for CP92 and cyan for CP98), and line types correspond to different shear rates in the perfusion chamber (continuous for a shear rate of 212 s^{-1}, and dashed for a shear rate of 1690 s^{-1}).

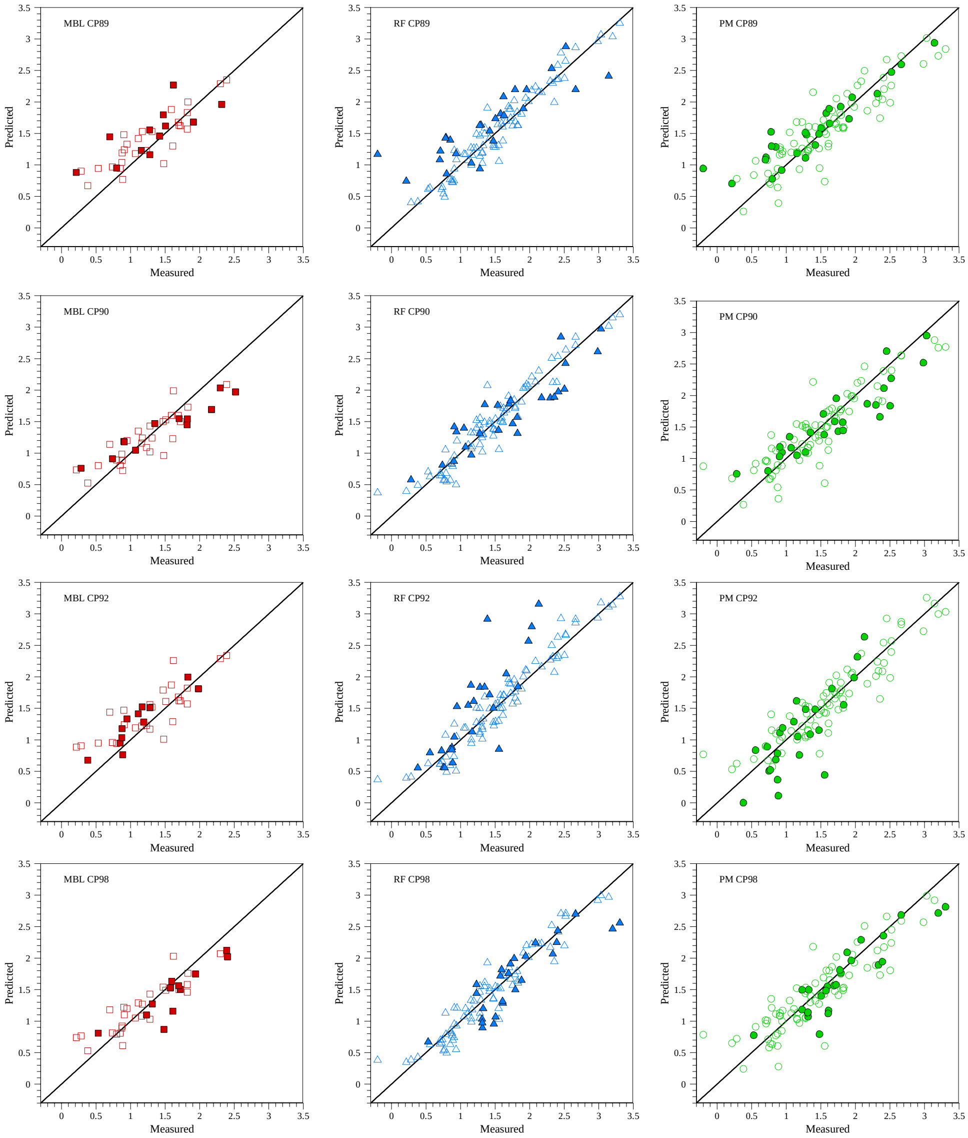

Figure S1- 5: Cross validation plot showing the platelet deposition predicted by the MBL model (red squares), RF (blue triangles) and the Phenomenological Model (green circles) for each individual pig. The train set is indicated with filled symbols and the test set with open symbols. The pig code indicates the pig used as test set. We show model predictions for the logarithm of the number of deposited platelets ($\log_{10}$ (number of platelets) cm^{-2} 10^{-6}) versus measured values of the same quantity.

S2 Multi-layer mass transfer boundary layer model (MBL)

Multi-layer deposition model

In our model, given a certain wall flux of platelets N_1'', platelets first form a monolayer on the substrate with a deposition rate that depends on the available deposition area, i.e. on the number of platelets P_1 already deposited as follows,

$$\frac{dP_1}{dt} = N_1''\delta W \left(1 - \frac{P_1}{P_\infty}\right) \tag{S2-1}$$

where

$$P_\infty = \frac{4\delta W}{\pi d_p^2}$$

is the maximum number of platelets in the monolayer, corresponding to 100% of coverage and $d_p = 2 \cdot 10^{-6}$m is the diameter of an adhered platelet [3].
Once platelets form a monolayer, we assume that platelets start to deposit on top of the monolayer at a rate

$$\frac{dP_2}{dt} = N_2''\delta W \left(\frac{P_1}{P_\infty}\right) \tag{S2-2}$$

Note that P_2 is the number of platelets deposited above the monolayer independently of the number of layers formed, so that the total number of platelets deposited at a given time is $P = P_1 + P_2$

Mass transfer boundary layer model for platelet fluxes

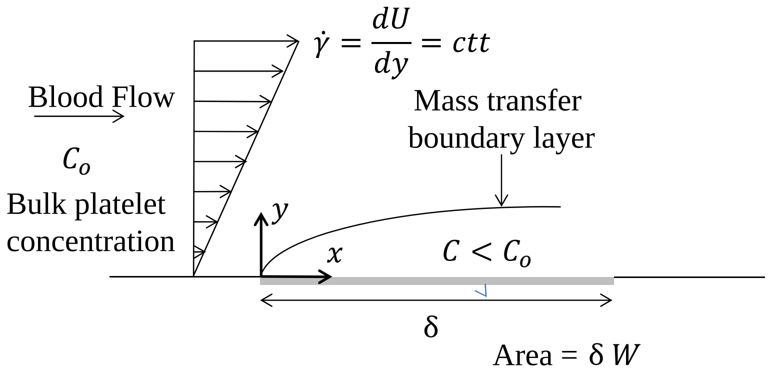

Figure S2- 1: Illustration of the mass transfer boundary layer model we use to predict platelet deposition numbers.

To model platelet fluxes, we follow a mass transfer boundary layer approach illustrated in Fig. S2-1. We assume that the substrate to which platelets adhere is regular, and generates a mass transfer boundary layer with a deficit of platelets. The concentration of platelets is a continuous variable that depends on two coordinates. We further assume that the flow is laminar and steady and the mass transfer boundary layer thickness is much thinner than the diameter of the perfusion chamber. Under these hypotheses the velocity gradient, or the shear rate γ, within the mass transfer boundary layer is constant (see Fig. S2-1) and platelets are transported towards the active portion of the wall, located at $0 \leq x \leq \delta$, by flow advection and diffusion.
Platelets are deposited on the active portion of the wall following a first order kinetic law,

$$P_{free} \xrightarrow{k} P_{deposited} \tag{S2-3}$$

According to this, a free platelet on the wall is transformed into an attached platelet.
The governing transport equation for the concentration of platelets can be written as,

$$\gamma y \frac{\partial C}{\partial x} = \frac{\partial^2 C}{\partial y^2} \tag{S2-4}$$

While Eq. S2-4 can be analytically solved for this particular set of the boundary conditions [1], [2] , the exact mathematical expressions for the concentration distribution and for the wall mass transfer rates are rather complicated. We therefore use the approximate expression for the surface averaged mass transfer rate when a first order chemical reaction occurs on a wall derived by

Pallares and Grau [4],

$$\frac{C_0}{N''} = \frac{1}{k} + \frac{C_0}{N''_C} \tag{S2-5}$$

where N''_C is the surface averaged mass transfer flux or for an infinite reaction rate, which produces a zero concentration of the reacting species at the wall. In this case the max flux [6],

$$N''_C = 0.8075 C_0 \left\{ \frac{\gamma D^2}{\delta} \right\}^{1/3} \tag{S2-6}$$

This expression of the surface averaged wall transfer rate is the conventional design relation for mass-transfer wall gauges used to measure the wall shear stress. If the mass flux N''_C is expressed using a convection mass transfer coefficient, K_C , as $N''_C = K_C C_0$, Equation S2-5 can be rewritten as,

$$N''_C = \frac{1}{\frac{1}{k} + \frac{1}{K_C}} C_0 = K_{eff} C_0 \tag{S2-7}$$

Equation S2-7 indicates that the effective convection mass transfer coefficient when a first order chemical reaction occurs on a wall, K_{eff}, can be understood as the inverse of the sum of the mass transfer resistance produced by the finite rate of the chemical reaction, $\frac{1}{k}$, and the resistance by convection in absence of reaction, $\frac{1}{K_C}$. The validity of Equation S2-7 is discussed in detail in [5]. The differences between Equation S2-7 and the analytical solution considering axial diffusion are less than 3% for $\frac{\gamma\delta^2}{D} > 3.2 \cdot 10^5$ and $\frac{k\delta}{D} > 10^5$. Considering the typically extreme values of the parameters in the perfusion experiments we analyze $\delta = 2.5 \cdot 10^{-2} m$, $D \approx 10^{-10} m^2 s^{-1}$, $\gamma = 1690 s^{-1}$ and $k \approx 10^{-4} ms^{-1}$ the previous quantities are $\frac{\gamma\delta^2}{D} \approx 10^{10}$ and $\frac{k\delta}{D} = 2.5 \cdot 10^4$ and the error of using Eq. S2-7 instead of the analytical solution is of about 0.5%. Under these conditions the boundary layer thickness is about $10^{-5} m$, two orders of magnitude smaller than the diameter of the perfusion chamber. Another important assumption in our model is the quasi steady state approximation for the platelet fluxes at the wall. The unsteady mass transfer boundary layer for an infinitely fast reaction, $k \longrightarrow \infty$, was considered analytically by Soliman and [5]. If the concentration of the reactive on the active portion of the wall is suddenly set to zero, the time needed for the wall mass flux to reach the steady state is $t \approx 0.8 \left(\frac{\delta^2}{\gamma^2 D} \right)^{1/3}$ which corresponds to about four seconds for experiments with $\delta = 2.5 \cdot 10^{-2} m$, $D \approx 10^{-10} m^2 s^{-1}$ and $\gamma = 212 s^{-1}$. [4] analyzed the time response of the mass transfer boundary layer for a finite reaction rate. According to the correlation given by these authors the time needed for the wall mass flux rate to reach the steady state is also about four seconds for $\delta = 2.5 \cdot 10^{-2} m$, $D \approx 10^{-10} m^2 s^{-1}$, $\gamma = 212 s^{-1}$ and $k \approx 10^{-4} ms^{-1}$. This time is much lower than the duration of the experiments, of several minutes, and consequently the quasi steady state approximation for the wall fluxes, adopted to obtain the rates at which platelets are deposited, is reasonable. According to Eq. S2-7, the instantaneous flux of platelets towards

the substrate to form the monolayer with an instantaneous length of $L = \delta\left[1-\left(\frac{P_1}{P_\infty}\right)\right]$ can be written as

$$N_1'' = \frac{C_0}{\frac{1}{k} + 1.238\left[\frac{\delta\left(1-\frac{P_1}{P_\infty}\right)}{\gamma D^2}\right]^{1/3}} \tag{S2-8}$$

Correspondingly, the instantaneous flux of platelets towards a layer of length of adhered platelets of $L = \delta\left(\frac{P_1}{P_\infty}\right)$ is

$$N_2'' = \frac{C_0}{\frac{1}{k_2} + 1.238\left[\frac{\delta\left(\frac{P_1}{P_\infty}\right)}{\gamma D^2}\right]^{1/3}} \tag{S2-9}$$

Introducing Eqs. S2-8 and S2-9 into Eqs. S2-1 and S2-2 leads to

$$\frac{dP_1}{dt} = \frac{C_0\delta W\left(1-\frac{P_1}{P_\infty}\right)}{\frac{1}{k_1} + 1.238\left[\frac{\delta\left(1-\frac{P_1}{P_\infty}\right)}{\gamma D^2}\right]^{1/3}} \tag{S2-10}$$

and

$$\frac{dP_2}{dt} = \frac{C_0\delta W\left(\frac{P_1}{P_\infty}\right)}{\frac{1}{k_2} + 1.238\left[\frac{\delta\left(\frac{P_1}{P_\infty}\right)}{\gamma D^2}\right]^{1/3}} \tag{S2-11}$$

respectively. Eqs. S2-10 and S2-11 constitute a set of ordinary differential equations with initial conditions $t = 0$, $P_1 = 0$ and $P_2 = 0$ with two unknown parameters k_1 and k_2. Eq. S2-10 can be directly integrated to obtain the time evolution of the deposited platelets on the substrate, while we solve Eq. S2-11 with a fourth-order Runge-Kutta method.
The integration of Equation S2-10 leads to

$$t\frac{\frac{DC_0}{\delta}}{\frac{P_\infty}{\delta W}} = \frac{-D}{k_1\delta} ln\left(1-\frac{P_1}{P_\infty}\right) + 3.7149\left(\frac{D}{\gamma\delta^2}\right)^{1/3}\left[1-\left(1-\frac{P_1}{P_\infty}\right)^{1/3}\right] \tag{S2-12}$$

where we have arranged the different terms to obtain the non-dimensional governing parameters of the problem. Note that $\frac{D}{k_1\delta}$ is the inverse of the Dämkholer number $Da = \frac{k_1\delta}{D}$ and $\frac{D}{\gamma\delta^2}$ is the inverse of the Péclet number based on the shear rate.
On the left hand side of Equation S2-12 the time scale is $\frac{\left(\frac{P_\infty}{\delta W}\right)}{\left(\frac{DC_0}{\delta}\right)}$, which is the maximum number of platelets per unit area $\frac{P_\infty}{\delta W}$, divided by a diffusion flux $\frac{DC_0}{\delta}$. According to this, the dimensionless time can be defined as $t^* = t\frac{\left(\frac{P_\infty}{\delta W}\right)}{\left(\frac{DC_0}{\delta}\right)}$
Equation S2-10 indicates that the rate at which the platelets are deposited on

the monolayer depends on a kinetic resistance $\frac{1}{k_1}$ and on a advection-diffusion resistance $R \propto \left(\frac{\delta}{\gamma D^2}\right)^{-1/3}$. Note that the length of the active portion of the wall, delta, affects differently both resistances. This has implications in the prediction of the deposition rate of platelets depending on the length of the damage of the vessel wall.

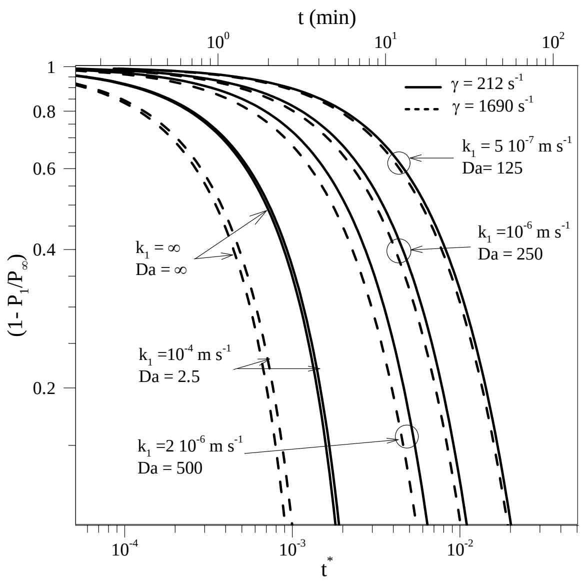

Figure S2- 2: Examples of time evolutions of the available area for the monolayer for different values of the kinetic constant and shear rates.

Figure S2-2 shows the fraction of available area of substrate, $1 - \frac{P_1}{P_\infty}$, as a function of time for different values of the kinetic constant k_1 and for typical parameter values in the perfusion experiments $\delta = 2.5 \cdot 10^{-2} m$, $W = 2.10^{-3} m$, $D \approx 10^{-10} m^2 s^{-1}$, $P_\infty \approx 10^7 pl$, $C_0 \approx 3 \cdot 10^{14} pl \cdot m^{-3}$ and $\gamma = 212 s^{-1}$ or , $\gamma = 1690 s^{-1}$. Note that a dimensional time scale in minutes has been included in the top horizontal axis of Fig. S2-2.
It is apparent that according to Eq. S2-10 and for the set of dimensional parameters considered in this example, the deposition of platelets to form the monolayer depends strongly on the kinetic constant. For $k_1 = 10^{-4} m \cdot s^{-1}$ the time evolutions are very similar to those corresponding to infinitely fast reaction kinetics, $k_1 \longrightarrow \infty$. In general, the shear rate increases the rate of deposition and this effect becomes more evident for the faster kinetics.

Estimation of the kinetic constants form empirical data

As we mentioned previously, Eqs. S2-10 and S2-11 constitute a set of ordinary differential equations with initial conditions $t = 0; P_1 = 0$ and $P_2 = 0$ with two unknown parameters k_1 and k_2 . In our analysis, in order to make predictions of the platelet deposition counts for a given set of test parameters,

we need to estimate first the kinetic constants using the training dataset.
In order to numerically determine the kinetic constants using the MBL model, we assume that k_1 depends only on the type of substrate used in the experiments. For each set of experiments with a given substrate, we then compute the time evolution of P_1 and P_2 (see Eqs. S2-10 and S2-11). We then perform the calculations for several values of k_1 and k_2 in the ranges $10^{-3} \leq k_1 \leq 10^{-8} m/s$. For each pair of values (k_1 , k_2), we then compute the absolute difference between the predicted value of the total number of platelets deposited and the corresponding experimental value at a given time. For each different substrate, we select the pair of values (k_1, k_2) that minimizes the absolute difference between the measured and predicted values. Note that this model cannot be directly applied to the cases with stenosis because in this case there is a strong the variation of the shear rate along the substrate.

S3 Calculation of shear rate in experiments with 80% of stenosis

Some experiments were performed using an experimental stenosis of 80%. We wanted to use this variable as a quantitative variable for our models (Random Forest and Phenomenological Model). In those cases we computed the shear rate solving numerically the Navier-Stokes equations in the three dimensional domain that emulate the perfusion chamber. For the numerical solution we used the finite volume CFD code FLUENT 14 (ANSYS Inc., Lebanon, USA). The simulation was computed under steady conditions. As boundary conditions we imposed: a) at inlet a parabolic velocity profile corresponding to the shear rate used in the ex-vivo experiments reported in Table 1, b) at outlet a constant pressure and c) at the wall a non-slip condition. We used the commercial CAD software ANSYS DesignModeler geometry tool (ANSYS Inc., Lebanon, USA) to reconstruct the virtual geometry and the computational mesh of the perfusion chamber with different stenosis. We performed a mesh dependence analysis of the average value of the wall shear rate "WSR" in the stenosis implementing three different structured meshes M1 (275000 cells), M2 (535000 cells) and M3 (750000 cells). The error relative to the WSR calculated with M3 between M2 and M3 was equal to 3%. For all calculations we used M2.

References

[1] Apelblat A. Mass transfer with a chemical reaction of the first order: Analyticalsolutions. *The Chemical Engineering Journal*, 19:19–73, 1980.

[2] Apelblat A. Mass transfer with a chemical reaction of the first order. effect of axial diffusion. *The Chemical Engineering Journal*, 23:193–203, 1982.

[3] Tokarev AA, Butylin AA, and Ataullakhanov FI. Platelet adhesion from shear blood flow is controlled by near-wall rebounding collisions with erythrocytes. *Biophysical Journal*, 100(4):799–808, 2011.

[4] Pallarès J and Grau FX. Mass transfer rate of a first-order chemical reaction on a wall at high Schmidt numbers. *International Journal of Heat and Mass Transfer*, 69:438–442, 2014.

[5] Soliman M and Chambré PL. On the time-dependent Lévêque problem. *International Journal of Heat and Mass Transfer*, 10(2):169–180, 1967.

[6] Lévêque MA. Les lois de la transmission de chaleur par convection. annales des mines. *Annales des Mines*, 13:201–299, 1928.
